# Bile acid: a potential inducer of colon cancer stem cells

**DOI:** 10.1186/s13287-016-0439-4

**Published:** 2016-12-01

**Authors:** Lulu Farhana, Pratima Nangia-Makker, Evan Arbit, Kathren Shango, Sarah Sarkar, Hamidah Mahmud, Timothy Hadden, Yingjie Yu, Adhip P. N. Majumdar

**Affiliations:** 1 Department of Veterans’ Affairs Medical Center, 4646 John R, Detroit, MI 48201 USA; 2 Karmanos Cancer Institute, Detroit, MI 48201 USA; 3 Department of Internal Medicine, Wayne State University, Detroit, MI 48201 USA

**Keywords:** Cancer stem cells, Colonospheres, ABCCB1, ABCG2, Deoxycholic acid, Lithocholic acid, Colonic epithelial cell, matrix metallopeptidases, Wnt/β-catenin signaling

## Abstract

**Background:**

Although the unconjugated secondary bile acids, specifically deoxycholic acid (DCA) and lithocholic acid (LCA), are considered to be risk factors for colorectal cancer, the precise mechanism(s) by which they regulate carcinogenesis is poorly understood. We hypothesize that the cytotoxic bile acids may promote stemness in colonic epithelial cells leading to generation of cancer stem cells (CSCs) that play a role in the development and progression of colon cancer.

**Methods:**

Normal human colonic epithelial cells (HCoEpiC) were used to study bile acid DCA/LCA-mediated induction of CSCs. The expression of CSC markers was measured by real-time qPCR. Flow cytometry was used to isolate CSCs. T-cell factor/lymphoid-enhancing factor (TCF/LEF) luciferase assay was employed to examine the transcriptional activity of β-catenin. Downregulation of muscarinic 3 receptor (M3R) was achieved through transfection of corresponding siRNA.

**Results:**

We found DCA/LCA to induce CSCs in normal human colonic epithelial cells, as evidenced by the increased proportion of CSCs, elevated levels of several CSC markers, as well as a number of epithelial–mesenchymal transition markers together with increased colonosphere formation, drug exclusion, ABCB1 and ABCG2 expression, and induction of M3R, p-EGFR, matrix metallopeptidases, and c-Myc. Inhibition of M3R signaling greatly suppressed DCA/LCA induction of the CSC marker ALDHA1 and also c-Myc mRNA expression as well as transcriptional activation of TCF/LEF.

**Conclusions:**

Our results suggest that bile acids, specifically DCA and LCA, induce cancer stemness in colonic epithelial cells by modulating M3R and Wnt/β-catenin signaling and thus could be considered promoters of colon cancer.

## Background

Bile acids, the normal component of the luminal content, are needed for absorption of lipids, cholesterol, and fat-soluble vitamins, and are considered to be the regulators of intestinal epithelial homeostasis in the gastrointestinal (GI) tract [1]. It has long been known that certain secondary bile acids, secreted into the intestinal lumen and involved in fat absorption, can promote colon carcinogenesis [2–4]. Levels of bile acids are known to differ as a result of patho-physiological and environmental conditions including obesity, genetic traits, and lifestyle [4–8].

Epidemiological studies have revealed that the incidence of colorectal cancer (CRC) among populations migrating from low-incidence to high incidence countries changes rapidly. Within one generation, the incidence of CRC reached its highest level in people migrating from Japan to Hawaii [9]. This has been thought to be largely due to changes in diet. This inference comes from the observation that large increases in both meat and fat-enriched diet are associated with a rise in CRC [10]. Additionally, populations with high incidence of colon cancer also show an increase in fecal concentrations of bile acids [2, 11, 12]. These observations suggest that increased exposure of the colonic lumen to high levels of bile acids may play a role in the natural course of development of CRC and certain bile acids have been categorized as potential tumor-promoting agents, particularly for colon cancer [13].

The roles of cancer stem cells (CSCs) in the maintenance and progression of many types of cancer are now well accepted and continue to gain credibility as more evidence is uncovered. The CSC model proposes that a population of CSCs representing a small fraction of cancer cells exists within the tumor. These cells are able to self-renew and are capable of initiating carcinogenesis and sustaining tumor growth [14]. CSCs are identified by specific surface epitopes. In CRC, CD133, CD24, CD44, CD166, EpCAM, and ALDHA1 are reported as CSC markers [15–18]. Cells expressing these surface epitopes have the ability to form tumors at a much diluted concentration in SCID mice that histologically resemble the primary tumor from which they were derived [19]. Several stages of carcinogenesis in colon cancer comprise subpopulations of CSCs, which are responsible for tumor cell transformation, growth, and proliferation. Recently, it has been reported that the expression CSC markers CD44 and CD166, associated with *KRAS* mutation in primary colonic tumors, represent a higher risk of lymph node involvement by the tumor and development of liver and lung metastasis [18].

However, little information is available about the intrinsic/extrinsic factor(s) that may stimulate the generation of CSCs in the colonic mucosa. We hypothesize that certain bile acids, specifically acid (DCA) and lithocholic acid (LCA), most notorious for their co-carcinogenic activity [20–22], may induce CSCs in colonic mucosal cells leading to the development of CRC. Studies were conducted to test this hypothesis.

## Methods

### Cell culture

Normal human colonic epithelial cells (HCoEpiC) were purchased from ScienceCell Research Laboratories (Carlsbad, CA, USA) [23]. HCoEpiC were generated from human colonic tissues, cryopreserved at passage one, and delivered frozen. HCoEpiC are negative for HIV-1, HBV, HCV, mycoplasma, bacteria, and fungi. They can be stimulated to express HLA class II and intercellular adhesion molecules in vivo [24]. They have also been shown to respond to a broad array of cytokines and exhibit growth characteristics [25].

All experiments were performed within 10 passages after obtaining the cell line. The cells were maintained in Dulbecco’s minimum essential medium (DMEM/F-12) supplemented with 10% fetal bovine serum (Invitrogen, Grand Island, NY, USA) and 1% gentamycin in a humidified incubator at 37 °C in an atmosphere of 95% air and 5% carbon dioxide.

#### mRNA quantitation

The cells, incubated with or without DCA or LCA, were subsequently treated with TRIzol reagent (Invitrogen, Carlsbad, CA, USA) as recommended by the manufacturer. RNA was isolated using the Rneasy Mini Kit (Qiagen).

For mRNA expression, cDNA was prepared with the SuperScript III First-Strand cDNA synthesis system for RT-PCR (Invitrogen) and analyzed in triplicate using the 2 × SYBR Green PCR Master Mix (Applied Biosystem) and the ABI Prism 7500 sequence detection system. PCR consisted of denaturation at 95 °C for 10 min and 40 cycles of 95 °C for 15 sec, 60 °C for 60 sec. Real-time qRT-PCR and analysis was performed in an Applied Biosystems 7500 Real Time PCR system. Ct values of mRNAs from each sample were calculated by normalizing with internal control β-actin. Each value represents the mean of three replicates.

The oligonucleotide primers were obtained from Integrated DNA Technology Inc. (Coralville, IA, USA). Matrix metallopeptidase (MMP) primers were the same as those reported by Xie et al. [26]. The primers for N-Cadherin, Slug, Twist, Vimentin, Zeb1, and Zeb2 were reported by Farhana et al. [27] and all other gene primers are presented in Table 1.

**Table 1:** Primer set for each gene

Gene	Sequences
*CD44*	Forward: 5′-AAGGTGGAGCAAACACAACC-3′
	Reverse: 5′-AACTGCAATGCAAACTGCAAG-3′
*CD166*	Forward: 5′-TAGCAGGAATGCAACTGTGG-3′
	Reverse: 5′-CGCAGACATAGTTTCCAGCA-3′
*KLF4*	Forward: 5′-CCCAATTACCCATCCTTCCT-3′
	Reverse: 5′-ACGATCGTCTTCCCCTCTTT-3′
*Nanog*	Forward: 5′-GATTTGTGGGCCTGAAGAAA-3′
	Reverse: 5′-CAGATCCATGGAGGAAGGAA-3′
*OCT4*	Forward: 5′-AGTGAGAGGCAACCTGGAGA-3′
	Reverse: 5′-GCCGGTTACAGAACCACACT-3′
*SOX2*	Forward: 5′-AACCCCAAGATGCACAACTC-3′
	reverse: 5′-GCTTAGCCTCGTGGATGAAC-3′
*c-Myc*	Forward: 5′-GGTGCTCCATGAGGAGACA-3′
	Reverse: 5′-CCTGCCTCTTTTCCACAGAA-3′
*EGFR*	Forward: 5′-CTTTCGATACCCAGGACCAAG-3′
	Reverse: 5′-CAACTTCCCAAAATGTGCCC-3′
*β-catenin*	Forward: 5′-TGGATGGGCTGCCTCCAGGTGAC-3′
	Reverse: 5′-ACCAGCCCACCCCTCGAGCCC-3′
*β-actin*	Forward: 5′-TCCTTCCTGGGCATGGAG-3′
	Reverse: 5′-AGGAGGGGCAATGATCTT-3′

### Fluorescence-activated cell sorting of CD44^+^CD166^–^ cells and spheroid formation

#### Isolation of CD44^+^CD166^–^ cells

All reagents and instrumentation used for flow cytometry were from BD Biosciences (San Jose, CA, USA). HCoEpiC were grown to 70–80% confluence, trypsinized, and then washed with sorting buffer (1 × PBS, 5% FCS). Cells were resuspended in 100 μl sorting buffer and stained with fluorophore-conjugated antibodies as follows: with anti-CD45-perCP-Cy5.5 (clone), anti-CD44-PECy7 (clone: G44-26), and anti CD166-PE (clone) or isotype-matched mouse IgG1-PerCP-Cy5.5, IgG2b-PE-Cy7, and PE-mouse IgG1 K (BD Pharmingen, San Diego, CA, USA). The stained cells were incubated for 1 h at 4 °C, subsequently washed with PBS, and resuspended in 0.5 ml sorting buffer. Compo-bead plus particles were stained in parallel, in accordance with the manufacturer’s instructions, to provide compensation controls. CD45^+^ cells were excluded from analysis as described previously [28]. Flow cytometry was performed on a FACS Vantage SE SORP and data were analyzed with CellQuest.

#### Formation of spheroids (colonospheres)

The sorted CD44^+^CD166^–^ cells from HCoEpiC were suspended in serum-free stem cell medium containing DMEM/F12 (1:1) supplemented with B27 (Life Technologies, Gaithersburg, MD, USA), 20 ng/ml EGF (Biomol International, Plymouth, PA, USA), 20 ng/ml fibroblast growth factor (Biomol International), and 100 μg/ml gentamycin. Approximately 150–200 cells/well were seeded in an ultra-low-attachment 96-well plate (Corning Inc., Lowell, MA, USA).

After 24 h, the cells were incubated in the absence (control) or presence of DCA or LCA for 12 days. Spheres formed were photographed and their size was measured utilizing an OLYMPUS CKX41 microscope supporting an Olympus DP72 digital camera and DP2-BSW software (Olympus Soft Imaging Solutions GmbH, Germany).

#### Statistical analysis

All statistics were performed using VassarStats web statistical software (Richard Lowry, Poughkeepsie, NY, USA). One-way analysis of variance (ANOVA) was performed to detect any differences between groups of control and bile acid-treated spheres. If the result of the ANOVA is significant (*P* < 0.01 vs control), pairwise comparisons between the groups were made by a post-hoc test (Tukey’s HSD procedure). The significance levels were set at *P* < 0.001, *P* < 0.01, and *P* < 0.05.

### Hoechst 33342 dye exclusion assay

HCoEpiC was treated with DCA and LCA (100 μg) for 30 days. Single-cell suspensions were washed with PBS (three times) and stained with Hoechst 33342 or H342 (5 μg/ml; Sigma Aldrich Inc., St Louis, MO, USA) for 45 min at 37 °C in Hank’s Balanced Salt Solution (HBSS) (Invitrogen, Carlsbad), vortexing gently every 15 min. The stained cells were collected, washed with PBS, and resuspended in 3 ml of PBS containing 2 μg/ml of propidium iodide, and subsequently analyzed under a fluorescent microscope under UV and bright field. The number of viable cells that excluded dye was calculated as described previously [29].

#### T-cell factor/lymphoid-enhancing factor luciferase assay

In order to determine the activation of Wnt/β-catenin signaling, the transcription factor T-cell factor/lymphoid-enhancing factor (TCF/LEF)-luc reporter plasmid (Qiagen) was used in HCoEpiC. The cells were transfected with Cignal TCF/LEF-Luc reporter plasmid (SA Biosciences, Frederick, MD, USA) using lipofectamine 3000. TCF/LEF transfected cells were treated with bile acids for 48 h after 24 h post transfection. Cells were harvested and analyzed for TCF/LEF activity using a luciferase assay kit (Promega-Biosciences, San Luis Obispo, CA, USA) according to the manufacturer’s instruction and the activity was measured on a BioTeK Synergy HT microplate reader.

#### Western blot and immunoprecipitation

For tyrosine phosphorylation studies of epidermal growth factor receptor (EGFR), the cells were grown in serum-free medium and treated with bile acids for 48 h, then lysed using lysis buffer (25 mM Tris-Cl (pH 8.0), 150 mM NaCl, 0.2% nonidet P-40, 10% glycerol 10 mM NaF, 8 mM glycerophosphate, 0.2 mM Na_3_VO_4_, 1 mM DTT, and 10 l/ml protease inhibitor cocktail; Sigma Aldrich).

For immunoprecipitation of protein(s), the cell lysates containing 0.3 μg protein were incubated with 1 μg of the anti-EGFR antibody (Santa Cruz Biotechnology) in a total volume of 500 μl lysis buffer overnight at 4 °C. Then 25 μl Protein G-Sepharose 4 Fast Flow beads (GE Healthcare Biosciences, Pittsburgh, PA, USA) added to each lysate and shaken for 2 h at 4 °C. EGFR immuno-beads were washed with lysis buffer containing protease inhibitors, and 20 μl loading buffer was added to each sample before boiling for 5 min. Western blot analyses were performed as described previously [30].

### Silencing of muscarinic 3 receptor

Human 4 unique 29mer shRNA duplexes targeting muscarinic 3 receptor (M3R), synthesized by Integrated Technology (IDT) using the TriFectamine kit, were utilized for silencing M3R. The gene silencing target sequences were from the coding sequence [PubMed:NM_000740] (CHRM3) and siRNA sequences, 5′-GTCATCAGCTTTGACAGATACTTTT-3′ (si-M3RT2) and 5′-GGGTCATTTCAATGAATCTGTTTACG-3′ (si-M3RT3). The scrambled sequences of siRNA were used as a control. M3R siRNA duplexes were transfected transiently in HCoEpiC using lipofectamine 3000 (Invitrogen). Bile acid (100 μM) was added after 24 h of post transfection and cells were harvested 72 h post transfection for mRNA quantitation and TCF/LEF activity.

### Immunofluoresence

For β*-*catenin immunostaining, HCoEpiC cells were grown on an eight-chambered slide overnight. DCA/LCA (100 µM) were then treated with cells for 24 h and incubated for 72 h. Cells were fixed with paraformaldehyde for 15 min, washed three times with PBS, and then cells were permealized with ice-cold methanol for 5 min and washed three times with PBS. Cells were blocked with 5% normal goat serum in PBS at room temperature for 1 h and then incubated with anti-β*-*catenin antibody (1:100 dilution; Cell Signaling) overnight at 4 °C. After washing with 1 × PBS, cells were incubated with anti-rabbit IgG–FITC conjugate (1:100 dilution) antibody for 30 min at room temperature. Cells were washed with PBS and then placed on cover slips with prolong gold antifade reagent containing DAPI (Cell Signaling Technology, Boston, MA, USA). Cells were photographed utilizing an Olympus microscope and an Olympus microscope digital camera with DP2-BSW software.

## Results

### Bile acid induction of CSCs

The primary objective of the current investigation is to determine whether bile acids, specifically DCA and/or LCA, would induce CSCs in colonic epithelial cells.

**Fig. 1:**
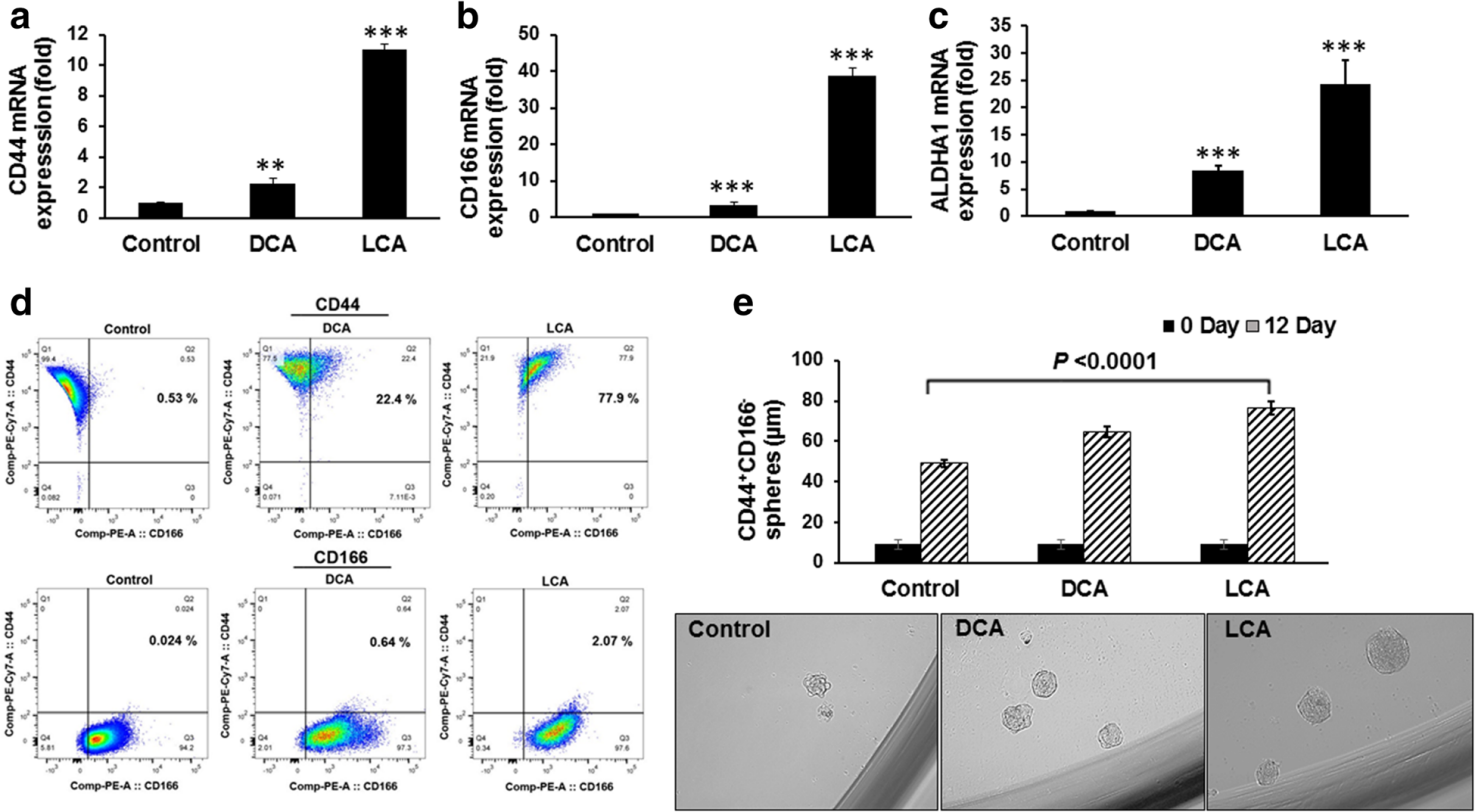
DCA and LCA induce increases in CSC markers. Quantitative real-time PCR showed significantly increased mRNA expression of CD44 (**a**), CD166 (**b**), and ALDHA1 (**c**) in HCoEpiC following incubation with 100 μM DCA or LCA for 72 h. Flow cytometric analysis of HCoEpiC showing an increased proportion of CD44-positive and CD166-positive cells following 12-day incubation in the absence (control) or presence of 100 μM DCA or LCA (**d**). DCA/LCA mediated the increase in spheroid formation by the CD44^+^CD166^–^ CSC phenotype of HCoEpiC (**e**); photomicrographs showing spheroids formed in response to DCA or LCA at the end of the 12-day incubation period. For spheroid formation, CD44^+^CD166^–^ cells were sorted by flow cytometry and 200–300 cells were seeded with B27 containing DMEM/F12 medium in 96-well low-attachment plates; 24 h after seeding, 100 μM DCA or LCA was added to the incubation medium, and cells were incubated for an additional 12 days. *0 Day*, cell size at the time of plating. The sizes of spheres were photographed and measured on a 100 μm scale at a magnification of 400× using an Olympus microscope. Data represent the mean ± standard deviation of 15 sphere determinations. **P < 0.01, ***P < 0.001, compared with control. Data were analyzed by ANOVA, Tukey HSD test for multiple comparisons (**e**). *DCA* deoxycholic acid, *LCA* lithocholic acid

In the first set of experiments, we examined the changes in expression of several CSC markers as well as the proportion of CSCs in HCoEpiC following exposure to DCA or LCA for 72 h. We observed that both DCA and LCA caused a marked 2-fold to 40-fold increase in the mRNA levels of CSC markers CD44, CD166, and ALDHA1 in HCoEpiC when compared with the control (Fig. 1a–c). However, LCA was found to be more effective than DCA in inducing the expression of these markers (Fig. 1a). In addition, the proportion of CD44-positive and CD166-positive populations was found to be markedly higher in DCA-treated or LCA-treated cells, compared with the corresponding vehicle-treated control (Fig. 1d). These observations suggest that DCA and LCA can induce CSCs in colonic epithelial cells. To further investigate this issue, we examined the ability of DCA and LCA to stimulate spheroid formation by HCoEpiC, a property common to both normal and CSCs. To conduct this investigation, we isolated the CD44^+^CD166^–^ phenotype from HCoEpiC by flow cytometry. This CSC phenotype was found to be a predictor of colonic adenoma [27, 28]. Cells were incubated after 12 days in stem cell media in the absence or presence of DCA or LCA. Incubation in stem cell media is known to kill epithelial cells but not stem cells/CSCs. Indeed, after 5–6 days of incubation, some cells began to form spheroids (surrogate tumors) in the absence or presence of bile acids. However, at the end of the 12-day incubation period, the size of the spheroids formed by the CD44^+^CD166^–^ CSC phenotype of HCoEpiC in the presence of DCA or LCA was found to be markedly greater than those formed in the absence of bile acids (controls) (Fig. 1e). At 12 days, the DCA-treated and LCA-treated spheroids also showed the required integrity of spheroids. These observations further suggest that bile acids induce stemness in normal human epithelial cells.

**Fig. 2:**
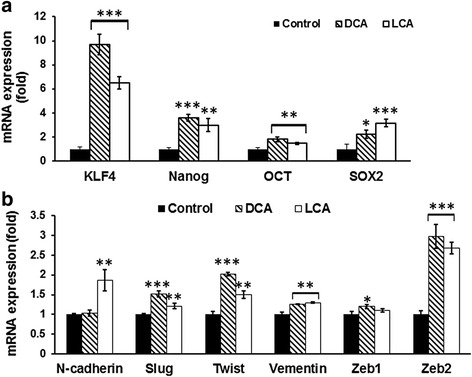
Exposure of DCA/LCA in HCoEpiC increased the expression of pluripotency genes. Levels of mRNA encoding the pluripotency genes *KLF-4, Nanog, OCT4*, and *SOX2* was significantly higher in cells incubated with DCA or LCA than control cells (**a**). Likewise, expression of EMT regulators N-Cadherin, Slug, Twist, Vimentin, Zeb1, and Zeb2 was also increased in response to 100 μM DCA or LCA (**b**). Results expressed as mean ± standard deviation of three separate experiments. *P < 0.05, **P < 0.01 and ***P < 0.001. *DCA* (deoxycholic acid), *LCA* (lithocholic acid)

Like normal stem cells, CSCs exhibit self-renewal in a de-differentiated state, pluripotency, but form tumors with a very small number of cells [31]. Four key transcription factors, OCT4 (POU class 5 homeo box 1), KLF4 (Kruppel like factor 4), SOX2 (SRY-box 2), and c-Myc (v-myc avian myelocytomatosis viral oncogene homolog) (OKSM), have been identified as pluripotency genes in CSCs. OKSM have been shown to induce dysplasia and tumorigenesis in vivo [31–36]. In view of this, we examined the expression of KLF4, Nanog, OCT4, and SOX2 in HCoEpiC in response to DCA/LCA. As has been observed for CSC surface epitopes, the expression of KLF4, Nanog, OCT4, and SOX2 was also significantly elevated following incubation for 7 days with 100 μM DCA or LCA, when compared with the corresponding control (Fig. 2a). These increases were accompanied by concomitant increases in the expression of N-cadherin, Slug, Twist, Vimentin, Zeb1, and Zeb2 (Fig. 2b) which are considered to be markers of epithelial–mesenchymal transition (EMT), cells that are thought to represent CSCs [37–39]. Taken together, the results suggest that DCA or LCA is able to transform colonic epithelial cells into CSCs.

**Fig. 3:**
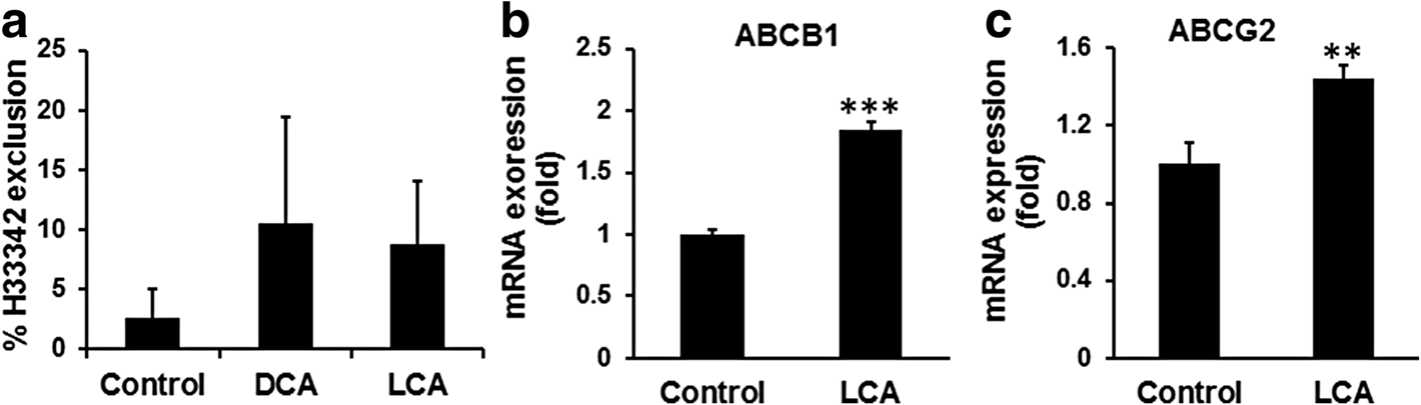
DCA/LCA induction of drug exclusion in HCoEpiC. Ability of HCoEpiC cells to exclude Hoechst dye (H33342) was greatly increased following 30-day incubation with DCA or LCA (**a**). Likewise, the expression of ABCB1 and ABCG2 was also increased in HCoEpiC cells following 18-day exposure to 50 μM LCA (**b**, **c**). Controls contained the appropriate vehicle. Data represent the mean ± standard deviation of three separate determinations. **P < 0.01 and ***P <0.001 Statistical significance determined by *t* test. *DCA* (deoxycholic acid), *LCA* (lithocholic acid)

To further determine whether DCA or LCA would indeed transform colonic epithelial cells to CSCs, the next set of experiments was conducted. One of the primary properties of CSCs, as opposed to adult stem cells, is their ability to resist chemotherapy because of increased drug efflux capacity [40]. We observed that the proportion of HCoEpiC that excluded Hoechst 33342 dye was greatly increased (2-fold to 3-fold) following 30 days of exposure to 100 μM of DCA or LCA, when compared with the control (Fig. 3a). One of the reasons for increased drug exclusion by cells is thought to be due to the elevated levels of ABC transporter proteins ABCB1 (ATP binding cassette subfamily B member 1) and ABCG2 (ATP binding cassette subfamily G member 2), members of the superfamily of ATP-binding cassette (ABC) transporters, whose primary function is to transport various molecules across the intracellular and extracellular membranes [40]. In HCoEpiC cells, we found LCA treatment for 18 days to cause a significant 1.5-fold to 2-fold increase in ABCB1 and ABCG2 mRNA levels when compared with the control (Fig. 3b, c). A similar phenomenon was also noted following 30-day exposure of HCoEpiC to DCA or LCA (data not shown). These results indicate the induction of CSCs in HCoEpiC following a prolonged exposure to the secondary bile acids, DCA or LCA.

**Fig. 4:**
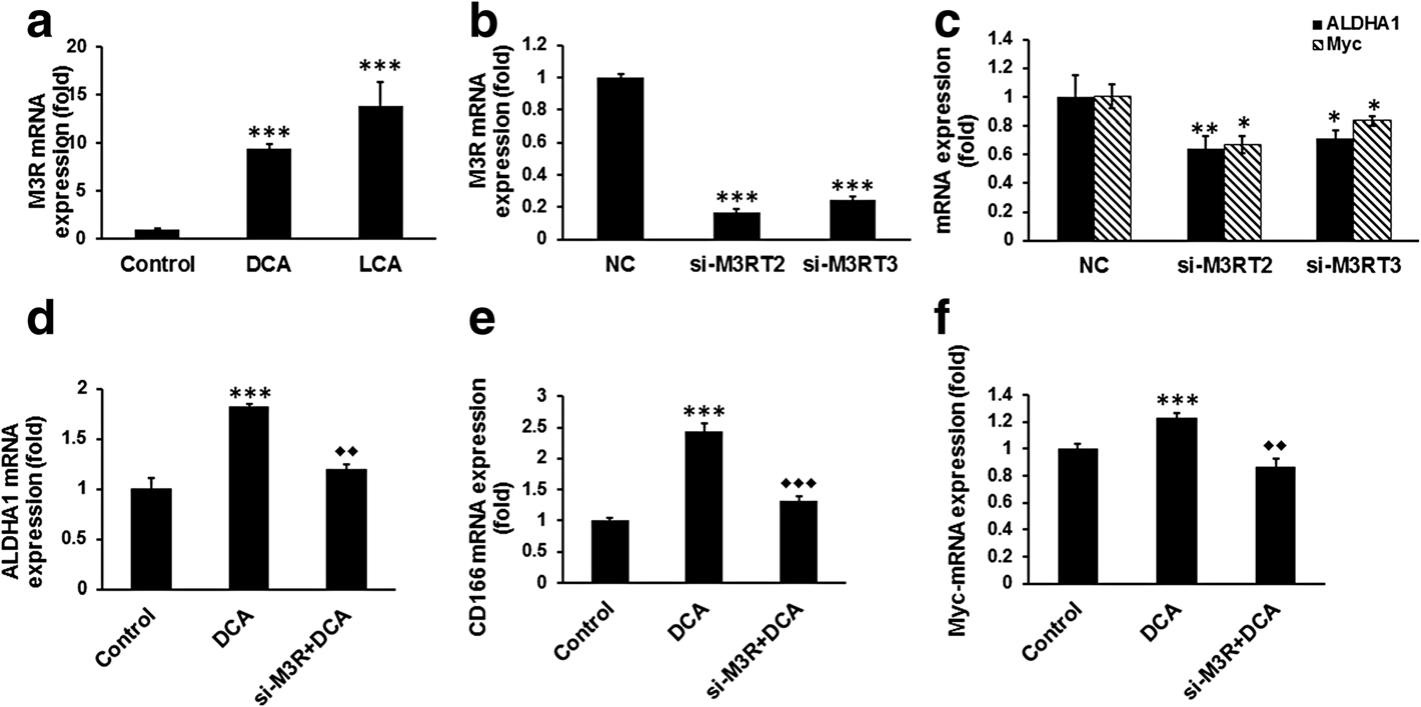
DCA/LCA induction of CSC phenotypic characters in HCoEpiC is mediated by M3R and knockdown of M3R decreased ALDHA1, c-Myc, and TCF/LCF activity in cells treated with DCA. Induction of M3R in HCoEpiC following incubation with 100 μM DCA or LCA after 72 h (**a**). Downregulation of M3R in cells following transfection with either of two siRNAs (si-M3RT2 and si-M3RT3) for M3R (**b**). ALDHA1 and c-Myc expression is reduced in M3R-downregulated cells (**c**). Suppression of DCA-induced stimulation of ALDH1, CD166, and c-Myc expression in M3R-downregulated cells (**d**–**f**). Data represent the mean ± standard deviation of three separate determinations. *P < 0.05, **P < 0.01, and ***P < 0.001, compared with the control. *DCA* (deoxycholic acid), *LCA* (lithocholic acid)

To further determine whether the presence of CSCs in HCoEpiC would lead to the processes of carcinogenesis, the following experiments were conducted. Earlier studies have reported that secondary bile acids such as LCA activate M3R and that this activation is important for colon cancer progression [41, 42]. In view of this, we examined the expression of M3R in HCoEpiC following exposure to DCA or LCA. We found DCA or LCA to induce an 8-fold to 12-fold increase in M3R mRNA levels in HCoEpiC, when compared with the control (Fig. 4a).

To further determine the role of M3R in DCA/LCA regulation of CSCs, we downregulated the receptor in HCoEpiC by the corresponding siRNAs. Two different siRNAs for M3R (si-M3RT2 and si-M3RT3) were utilized. Transfection of HCoEpiC with either si-M3RT2 or si-M3RT3 resulted in a marked 80–85% reduction of the receptor (Fig. 4b), accompanied by a significant reduction in the expression of ALDHA1 and c-Myc, when compared with the corresponding control (Fig. 4c). Additionally, we observed that while DCA caused a significant (100–300%) increase in the expression of ALDHA1, CD166, and c-Myc in HCoEpiC, no such increase by DCA could be observed in cells when M3R was downregulated (Fig. 4d–f).

**Fig. 5:**
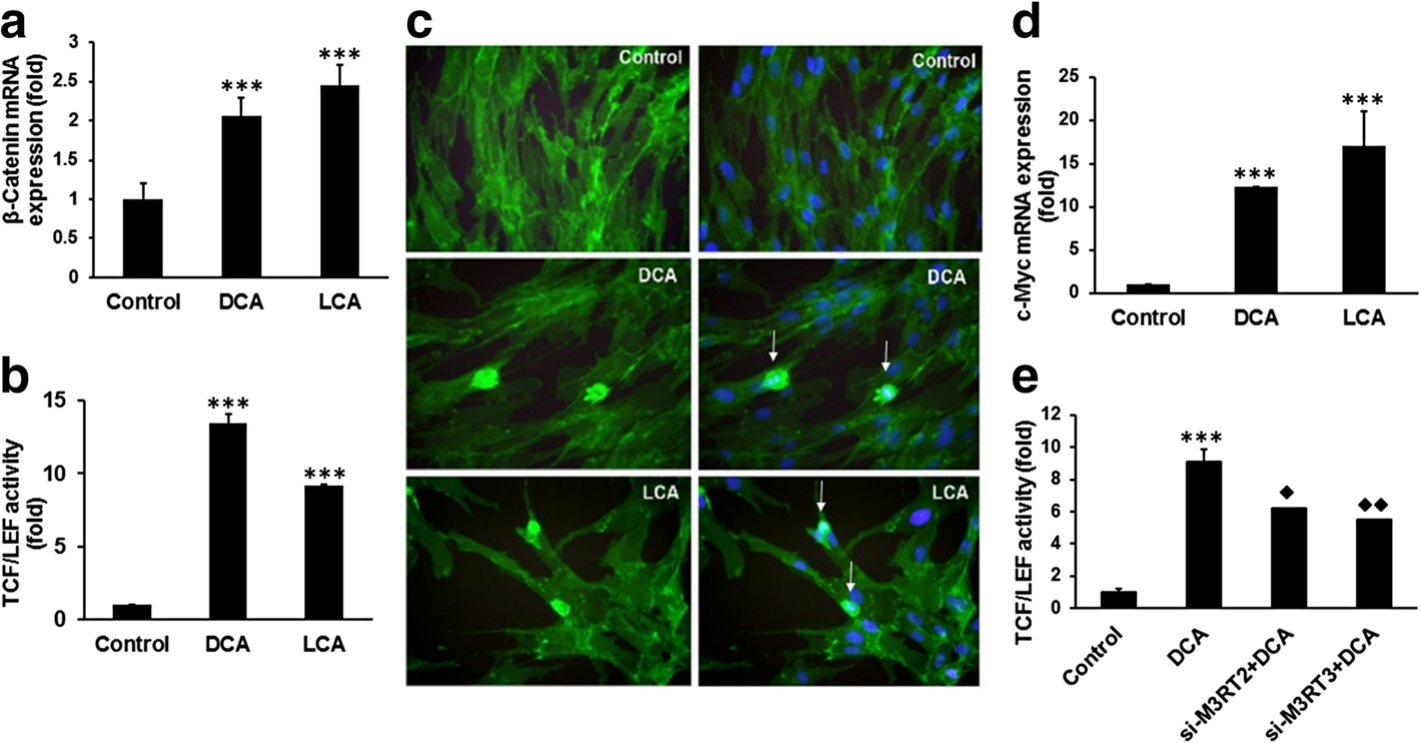
Bile acids induce Wnt-β-catenin signaling pathways and increase the expression of the target gene c-Myc in HCoEpiC. Real-time qPCR showing an increased expression of β-catenin mRNA in HCoEpiC cells following 72-h incubation in the presence of 100 μM DCA or LCA (**a**). Induction of transcriptional activity of TCF/LEF in HCoEpiC in response to 100 μM DCA or LCA treatments for 72 h (**b**). Photomicrographs showing increased nuclear localization of β-catenin in HCoEpiC following 72 h incubation with 100 μM DCA or LCA; controls were incubated with an equivalent volume of the vehicle (**c**): *left panel* , β-catenin immunostained cells; *right panel*, merged photograph of β-catenin and nucleus stained with DAPI; *arrow*, nuclear localization of β-catenin in cells. Increased expression of c-Myc in HCoEpiC following 72-h exposure to 100 μM DCA or LCA (**d**). DCA/LCA-mediated induction of transcriptional activity of TCF/LEF is greatly suppressed in M3R-downregulated HCoEpiC (**e**). Cells were photographed on a 100 μm scale at a magnification of 400×. Results expressed as mean ± standard deviation of three separate experiments. *P < 0.05, **P < 0.01 and ***P < 0.001. *DCA* (deoxycholic acid), *LCA* (lithocholic acid), *TCF/LEF* (T-cell factor/lymphoid-enhancing factor). Diamonds represent significant reduction in M3R down-regulated cells in response to DCA compared to those without siM3R transfected cells

Wnt/β-catenin signaling plays a pivotal role not only in maintaining homeostasis of intestinal crypt but also in regulating proliferation of colon CSCs [43–45]. We have observed that in HCoEpiC, DCA, and LCA not only stimulated the expression of β-catenin, but also the Wnt/β-catenin signaling, as evidenced by the induction of transcriptional activity of TCF/LEF (Fig. 5a, b).

The Wnt/β*-*catenin signaling pathway leads to de-phosphorylation, stabilization, and nuclear translocation of β-catenin. Nuclear β*-*catenin forms a complex with TCF/LEF family transcription factors and acts as a coactivator to express target genes in canonical Wnt signaling pathway such as CCND1 and MYC [46, 47]. We found in HCoEpiC that exposure to DCA/LCA resulted in a marked 2-fold to 3-fold increase in the expression of β-catenin, accompanied by a marked induction of transcriptional activity of TCF/LEF, when compared with the corresponding controls (Fig. 5a, b). We have also observed increased nuclear localization of β-catenin in HCoEpiC following exposure to DCA or LCA (Fig. 5c). These increases were also accompanied by a 12-fold to 15-fold increase in the level of c-Myc (Fig. 5d), one of the downstream effectors protein of Wnt/β-catenin signaling [47–49]. c-Myc also regulates stemness in CSCs [31]. In the presence of si-M3R-RNA, DCA-mediated stimulation of transcriptional activity of TCF/LEF was decreased by about 45% (Fig. 5e), indicating a role for M3R in regulating bile acid induction of carcinogenesis in colonic epithelial cells.

**Fig. 6:**
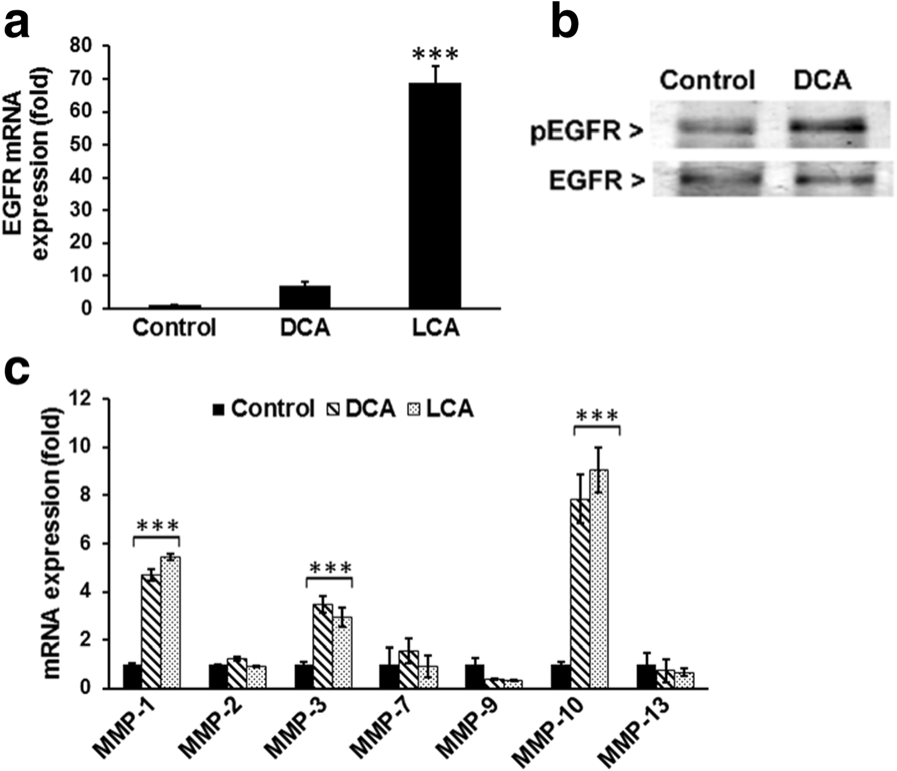
DCA/LCA increased the expression and activation of EGFR in HCoEpiC. Real-time qPCR showing increased expression of EGFR mRNA in cells in response to DCA and LCA (**a**). Western blot analysis indicates increased tyrosine phosphorylation (Y992) of EGFR in response to DCA (**b**). Real-time qPCR showing changes in the expression of the MMPs in cells in response to DCA or LCA (**c**). Results represent the mean of three separate determinations ± standard deviation. ***P <0.001, compared with control. *DCA* (deoxycholic acid), *EGFR* (epidermal growth factor receptor), *LCA* (lithocholic acid), *MMP* (matrix metallopeptidase)

Muscarinic agonist-induced cell proliferation is mediated by cross-talk between high expression of M3R and EGFRs in human colon cancer cells and M3R activation causes a marked enhancement in MMPs [50, 51]. We also found a similar phenomenon in HCoEpiC; DCA and LCA not only produced a marked increase in the expression of EGFR but also greatly activated EGFR, as determined by tyrosine phosphorylation (activated form; measured using ^Tyr^992EGFR antibody) (Fig. 6a, b). This increase was associated with a marked rise in MMP mRNAs, MMP1, MMP3, and MMP10 mRNA in HCoEpiC (Fig. 6c).

## Discussion

Higher levels of secondary bile acids in feces and an increased incidence of colorectal cancer (CRC) were observed in patients that consumed high-fat/low-fiber diets [52–54]. Several epidemiological studies have also revealed an association between bile acids and CRC. The presence of CRC has been associated with a higher fecal LCA/DCA ratio and with elevated fecal secondary bile acids levels [53–56]. Although the precise mechanism(s) by which bile acids induce colon carcinogenesis is poorly understood, DCA and chenodeoxycholic acid (CDCA)-mediated genotoxicity in normal human colonic epithelial cells as well as in tumor cells has been found to be due to DNA oxidative damage [57]. Increased apoptosis and other forms of toxicity have been observed in the liver following prolonged exposure to high levels of bile acids [58–61].

The hydrophobic bile acids DCA and LCA appear to be the most significant bile acids with respect to the development of CRC [5]. Data from several in-vitro and in-vivo studies also support this contention. Exposure of normal colonic epithelial cells to DCA has been shown to cause mitotic aberrations that are precursors of aneuploidy and are indicators of genome instability [6].

Our current observation that the proportion of CSCs as well as the expression of several markers of CSCs, formation of spheroids (colonospheres), and the levels of pluripotency markers KLF4, Nanog, OCT4, and SOX2 and EMT markers such as Vimentin, Slug, Twist, and Zeb2 are greatly augmented in HCoEpiC in response to DCA or LCA suggests that these secondary bile acids induce the formation of CSCs. Further support for this inference comes from the observation that drug exclusion, an inherent property of CSCs as determined by exclusion of H33342, is also markedly augmented in normal human colonic epithelial cells following prolonged exposure to DCA or LCA. The fact that the expression of multiple drug resistance (MDR) transporter genes ABCB1 and ABCG2 is also augmented by DCA and LCA provides additional support to our contention that the secondary bile acids, specifically DCA and LCA are able to induce transformation of normal colonic epithelial cells to CSCs, which are known to play a pivotal role in the development and progression of many malignancies, including CRC [62].

Bile acid-mediated induction of colon carcinogenesis appears to be regulated by the muscarinic cholinergic family of G-protein-coupled receptor, which consists of five subtypes M1R–M5R. Results from earlier studies suggest that secondary bile acids such as LCA activate M3R and that this activation is important for colon cancer progression [41, 42]. In the current investigation, we have also found M3R expression to be greatly elevated in response to bile acids and that this increase is accompanied by a concomitant rise in the expression of MMP-1, MMP3, and MMP10 mRNA in HCoEpiC. MMPs are not only important in maintaining extracellular homeostasis but also play a prominent role in cancer cell invasion. Overexpression of MMP1, MMP2, MMP3, MMP7, MMP9, and MMP13 correlates with worse outcomes in cancer patients [26, 63–66].

Our observation that DCA or LCA could stimulate the expression of M3R in normal human colonic epithelial cells and that this induction is greatly attenuated when M3R is downregulated in colonic epithelial cells further supports a role for M3R in regulating DCA/LCA-mediated modulation of colon carcinogenesis. Additional support for this inference comes from the observation that DCA-mediated induction of Wnt/β-catenin signaling in M3R-downregulated colonic epithelial cells, a prerequisite for colon carcinogenesis [67, 68], is greatly diminished.

## Conclusions

Taken together, our current data show that certain secondary bile acids, specifically DCA and LCA, which are known for their co-carcinogenic activity, are able to induce CSCs in colonic epithelial cells. This is evidenced by the observation that DCA or LCA are not only able to stimulate the expression of several surface epitopes of colon CSCs but also the markers of EMT. These increases are associated with increased spheroid formation, a rise in the expression of multiple drug resistance transporter genes, and drug exclusion. DCA/LCA induction of colon carcinogenesis is found to be regulated by M3R.
